# *Echinococcus multilocularis* and Other Taeniid Metacestodes of Muskrats in Luxembourg: Prevalence, Risk Factors, Parasite Reproduction, and Genetic Diversity

**DOI:** 10.3390/pathogens11121414

**Published:** 2022-11-24

**Authors:** Matilde Martini, Sonja Dumendiak, Anna Gagliardo, Francesco Ragazzini, Letizia La Rosa, Dimitri Giunchi, Frank Thielen, Thomas Romig, Alessandro Massolo, Marion Wassermann

**Affiliations:** 1 Ethology Unit, Department of Biology, University of Pisa, 56126 Pisa, Italy; 2 Parasitology Unit 190p, Institute of Biology, University of Hohenheim, 70599 Stuttgart, Germany; 3 Telethon Institute of Genetics and Medicine (TIGEM), 80078 Pozzuoli, Italy; 4 Neuroscience and Brain Technologies Department, Istituto Italiano di Tecnologia, 16163 Genoa, Italy; 5 natur&ëmwelt Fondation Hëllef fir d‘Natur, 9753 Heinerscheid, Luxembourg; 6 Center of Biodiversity and Integrative Taxonomy, University of Hohenheim, 70599 Stuttgart, Germany; 7 UMR 6249 CNRS, Chrono-Environnement, Université Bourgogne Franche-Comté, Campus La Bouloie, 25030 Besançon, France; 8 Faculty of Veterinary Medicine, University of Calgary, Calgary, AB T2N 4Z6, Canada

**Keywords:** *Echinococcus multilocularis*, *Taenia*, *Hydatigera*, *Versteria*, muskrat, Luxembourg, epidemiology, reproduction, haplotype

## Abstract

Muskrats (*Ondatra zibethicus*) are competent intermediate hosts for *Echinococcus multilocularis*, are frequently infected with this zoonotic cestode, and have even been proposed as a target species to monitor endemicity levels of this parasite. However, their contribution to maintaining the parasitic lifecycle is still unclear. To obtain data on infection frequency and reproductive potential, 280 muskrats from the Grand Duchy of Luxembourg were examined for cestode larvae in the years 2013–2017. Based on morphological and molecular identification, *Echinococcus multilocularis* was found at a prevalence of 14.6%. Other metacestodes were *Hydatigera kamiyai,* with a prevalence of 45.7%, *Taenia martis* with 8.9%, *Taenia polyacantha* with 5.0%, and *Versteria mustelae*, which was found in 0.7% of all muskrats. More than 80% of *E. multilocularis*-infected muskrats contained fertile metacestodes with a mean number of >300,000 (and up to 1,609,816) protoscoleces, which is by far the highest reproductive potential known from any intermediate host species in Europe. Temporal analysis of *E. multilocularis* prevalence within the study period (and in comparison with earlier data) strongly indicates a robust increase in the studied area. Host age seemed to be an important risk factor for infection, as well as co-infections with *Hydatigera kamiyai*. A preference for the right medial lobe of the liver as the location of *E. multilocularis* metacestode was observed. Intraspecific genetic variation among 89 discrete *E. multilocularis* metacestodes was non-existent based on 300–1590 bp sections of *cox1*. This is a stark contrast to *H. kamiyai*, of which nine haplotypes were found on a short 318 bp section of *cox1*, resulting in genetic diversity in the small country of Luxembourg at a similar level than previously reported from large stretches of Europe and northern Asia.

## 1. Introduction

Human alveolar echinococcosis (AE) is a zoonosis caused by the tumor-like larval stage of the taeniid cestode *Echinococcus multilocularis*. AE is considered one of the world’s most lethal parasitic diseases, with an estimated >18,000 new cases per year, and is recognized by the World Health Organization as a neglected zoonotic disease that requires multidisciplinary research efforts to understand its complex transmission system [1].

The adult stages of *E. multilocularis* live in the intestinal lumen of the definitive host (DH), which is a carnivore (usually a canid). After the release of the terminal proglottids containing 200–300 eggs into the environment with the host feces, various species of intermediate hosts (IH), usually rodents, become infected by accidental ingestion of eggs present in the environment. In the host intestine, the eggs release the first larval stage (oncosphere), which usually migrates to the liver through the bloodstream. In the liver, the parasite develops into the second larval stage (metacestode), which continues to grow as a solid space-occupying mass consisting of numerous microcysts. Eventually, protoscoleces are formed inside the microcysts. After the predation of an infected IH, protoscoleces develop into adult worms in the small intestine of the definitive host. Humans are accidental hosts who do not take part in the life cycle of the parasite and who, upon ingestion of eggs, develop metacestodes (usually in the liver) whose continuous growth causes the progressive replacement of the organ tissue, hence inducing severe pathological conditions and high mortality when left untreated [1].

In Europe, *E. multilocularis* mainly circulates between the red fox (*Vulpes vulpes*) as DH and several species of Arvicolinae as IHs. The common vole (*Microtus arvalis*) and the montane water vole (*Arvicola scherman*) are often considered the most important IH species in temperate Europe, although the contribution of other arvicoline species is not fully understood [2].

One such species is the muskrat (*Ondatra zibethicus*), a semi-aquatic rodent that is native to North America and was introduced to various parts of the world in the early 1900s as a fur animal. In Europe, the first introduction was apparently in 1905 near Prague, followed by numerous intentional releases, escapes from fur farms, and intra-European translocations in various countries [3]. At present, muskrats are distributed all over Europe with the exception of the Iberian Peninsula, Italy, Greece, Britain, and Ireland (eradicated); in suitable habitats, population densities may exceed 50 animals per ha (lit. in [4]). Muskrats are preyed on by a variety of carnivores, including red foxes; in parts of Europe where the invasive American mink (*Neovison vison*) is spreading, muskrat numbers seem to decline [5,6].

The role of muskrats in the lifecycle of *E. multilocularis* is not yet well understood. They seem to be more frequently infected than other suitable IH species in the same region, and prevalence in central Europe may be as high as 39%; metacestodes are highly fertile and may reach a massive size in some individuals [7,8]. Conversely, their restriction to aquatic habitats and lower population sizes certainly decreases the probability of muskrats as infection sources for foxes [2].

Apart from *E. multilocularis*, muskrats in Europe are suitable hosts for the larvae of at least five other species of taeniid cestodes [9]. Two of these, *Taenia crassiceps* and *T. polyacantha*, have similar host ranges as *E. multilocularis* with the red fox as the principal DH, whereas *T. martis*, *Versteria mustelae,* and *Hydatigera* spp. typically use mustelids or cats as DH. According to the few surveys that have previously addressed the frequency of these parasites in muskrats, the ubiquitous cat tapeworm (*Taenia taeniaeformis*) was the most frequent parasite, with approximately 20–50% of muskrats infected in various parts of Germany and the Netherlands [9,10,11,12,13]; in southeastern Belgium, bordering Luxembourg, even 65.8% of 657 muskrats were found infected [14]. *Taenia taeniaeformis* was later transferred to the genus *Hydatigera* [15] and was recently split into distinct clades, of which the genetic and morphological variant, which is most frequent in central Europe, was described as *H. kamiyai* [16].

Like surrounding regions, Luxembourg is known as an endemic area for *E. multilocularis*; a prevalence in foxes of 23.8% (based on 84 examined animals) was reported in 2018 [17]. No published data are available for IH infection with *E. multilocularis* in this country. For the present study, trapped muskrats were obtained from two distinct river habitats in northern Luxembourg, where the muskrat population is routinely controlled to protect populations of freshwater pearl mussels (*Margaritifera margaritifera*) and thick-shelled river mussels (*Unio crassus*) (LIFE Resto-unio project). The aims of this study were (1) to estimate the prevalence of *E. multilocularis* and other taeniids, (2) to identify possible risk factors for infection, (3) to assess parasite reproductive potential, (4) to verify the hypothesis of a lobe preference for the metacestodes settlement and their development in the liver and, and (5) to assess the genetic diversity of *E. multilocularis* and *Hydatigera* spp. with a focus to obtain more data on the identity of *Hydatigera* spp. in central Europe.

## 2. Materials and Methods

### 2.1. Study Site

**Figure 1:**
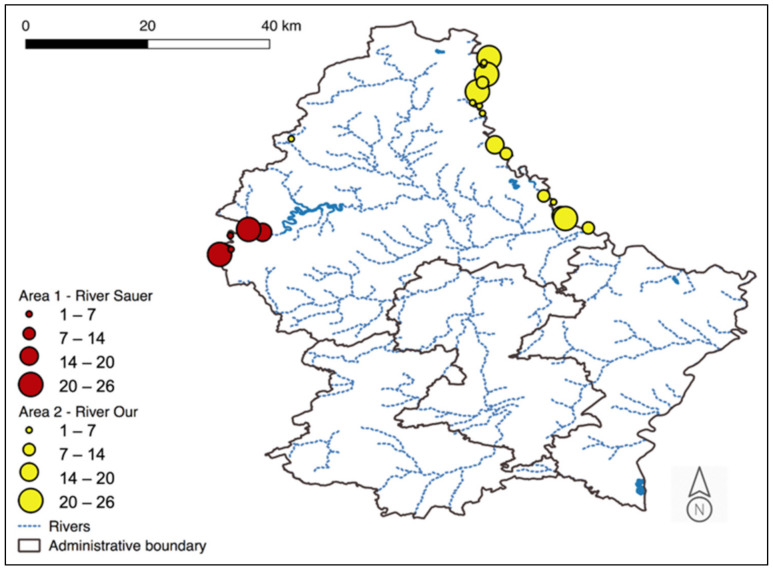
Collection sites of muskrats along the Sauer and Our River in the Grand Duchy of Luxembourg. Dots represent the trap sites; dot sizes indicate the numbers of trapped muskrats.

From December 2013 to November 2017, a total of 280 muskrat were trapped and killed in two rural areas of northern Luxembourg along the rivers Sauer and Our (Figure 1). Each carcass was labeled with date and precise trapping site and stored at −20 °C until examination.

### 2.2. Necropsy

At necropsy, sex and weight were determined, and the carcasses were visually searched for metacestodes in the abdominal and thoracic cavities as well as subcutaneously and inside organs. The liver was removed, and each lobe (left lateral, left medial, right medial, right lateral, and caudate) was separated and individually weighed and superficially searched for metacestodes; hepatic tissue was then cut into slices, squeezed between glass slides, and inspected under a microscope. Lesions were counted and preliminarily allocated to taeniid species. In the case of *E. multilocularis*, all lesions were weighed and further processed to determine the number of protoscoleces. All lesions were stored at −20 °C for subsequent molecular identification.

### 2.3. Protoscoleces Count

To estimate the number of protoscoleces of *E. multilocularis*, samples of metacestode tissue were removed from each lesion. In the case of lesions weighing < 1 g, the whole lesion was analyzed; with lesions weighing from 1 g to <10 g, one 1 g aliquot was analyzed; and with lesions weighing > 10 g, two 1 g aliquots from different parts of the metacestode were analyzed. The 1 g aliquots were then homogenized in 1 mL of water and passed through a nylon mesh of 1 mm pore size using a pestle. The solution was diluted with water, and the protoscoleces were counted under 50× magnification. The total number of protoscoleces in each lesion was extrapolated from the aliquots.

### 2.4. Data Analyses

Animal age was estimated using the weight as a proxy [3] and grouped into juveniles (<600 g), subadults (600–1000 g), and adults (>1000 g).

The collected carcasses were then grouped according to their river basin of origin (Sauer and Our) and to the period of collection (from 2013 to 2015 and 2016/2017).

Univariate analysis [18] was preliminarily performed to identify potential risk factors for *E. multilocularis* and the distribution of lesions in the different liver lobes. Pearson’s Chi-square test with a simulated p-value according to the Monte Carlo method based on 2000 replicates was used to study the relationships between the presence of infection and risk categories (area, season, sex, period, age, and co-infections by the other cestodes) and to further evaluate the distribution of *E. multilocularis* lesions in different liver lobes [19,20].

To model the probability of *E. multilocularis* infection in muskrats, we formulated a binomial generalized linear model [21] with a logit link using as risk factors the geographic origin of collection (Sauer, Our), season of capture (winter, spring, summer, autumn), period (from 2013 to 2015, 2016 and 2017), sex, age class, and other taeniid co-infections (i.e., presence or absence of metacestodes of other species).

A simple linear model (LM) approach was used to test the hypotheses that the density of lesions and the density of protoscoleces differed between different lobes, using the lobes as fixed factors and the densities as outcome variables in two different models [21].

Furthermore, a binomial mixed-effects model with a logit link [21] was formulated to test the hypothesis of a lobe preference for the metacestodes settlement and their development in the liver. The presence of lesions in the liver lobes was treated as binary response variable, whereas each lobe per animal was settled as an independent fixed variable. Considering that each subject contained a group of five values (one per lobe), we set muskrat ID as a random factor [22].

All statistical analyses were performed using R statistical software version 3.5.1 [23].

To run the univariate analysis, *chisq*.*test* function was used, and in the case of the presence of lesions in the five liver lobes, we used the same function with *simulate.p.value* (package *stats*).

The first linear models (LM) were formulated by using the *lm* function, whereas the binomial generalized linear model with a logit link was formulated using the *glm* function in the package *stats*. A general linear mixed-effects model was developed using the *glmer* function in the package *lme4* [24].

Both models were confronted with their null models using ANOVA function (package *stats*).

Finally, prevalence confidence intervals (CIs) were estimated using Wilson 95% CI implemented in EpiTools (http://epitools.ausvet.com.au/; accessed on 25 February 2021) [25].

### 2.5. Molecular Analysis

For confirmation of *E. multilocularis*, DNA was obtained from metacestode material. Small pieces of metacestode tissue were transferred to 20 µL of 0.02 M NaOH solution and lysed for 10 min at 95 °C [26]. The target sequence for DNA amplification was the complete mitochondrial *cox1* gene (1608 bp). A nested PCR was performed using the primers *cox1*_for 5′-GTG GAG TTA CTG CTA ATA ATT TTG-3′ and *cox1*_rev 5′-TAC GAC TYA CTT AT CAC-3′ for the first PCR and the primer pair *cox1*_fornest 5′-TTA CTG CTA ATA ATT TTG TGT CAT-3′ and *cox1*_revnest 5′-GCA TGA TGC AAA AGG CAA ATA AAC-3′ for the nested PCR [27,28].

For the identification of other cestodes, a 466 bp fragment of the *cox1* gene was amplified. For this purpose, non-specific primers were designed that amplify the target sequence of a broad spectrum of cyclophyllidean cestodes (Dumendiak, unpublished): forward 5′-TTT GAT CGT AAA TTT AGT TCT GC-3′ (Cyclo_F), reverse 5′-GCA ACA ACA AAT CAA GTA TCA TG-3′ (Cyclo_R), nested forward 5′-GTT CTG CTT TTT TTG ATC C-3′ (Cyclo_nF) and nested reverse 5′-GTA TCA TGT AGA ACT TTA TC-3′ (Cyclo_nR).

All PCRs were performed in a 50 µL reaction mixture containing 10 mM Tris–HCl (pH 8.3), 50 mM KCl, 2 mM MgCl_2_, 20 pmol of each respective primer, 0.2 mM dNTPs, 1.25 U of Ampli-Taq Polymerase (Applied Biosystems, Darmstadt, Germany) and 1 µL of lysate in the first PCR. In the nested PCR 1 µL of the first amplification product was used as template DNA. For the *E. multilocularis* PCR, the condition during amplification was as follows: initial denaturation for 5 min at 95 °C, 35 cycles of denaturation at 95 °C for 30 s, annealing at 52 °C during the first PCR, and 55 °C during nested for 30 s and elongation at 72 °C for 2 min, followed by a final elongation for 5 min at 72 °C. The condition for the amplification of other cestodes was initial denaturation for 5 min at 95 °C, 35 cycles of denaturation at 95 °C for 30 s, annealing at 50 °C for first and nested PCR for 30 s, and elongation at 72 °C for 1 min, followed by a final elongation for 5 min at 72 °C. All amplification products were purified with a High Pure PCR Product Purification Kit (Roche, Mannheim, Germany), following the instructions of the manufacturer. The sequencing of each purified product was performed by GATC (Konstanz, Germany). The obtained DNA sequences were compared with existing sequences in GenBank databases using the NCBI BLAST search tool (www.blast.ncbi.nlm.nih.gov).

Sequences of *H. kamiyai* were further screened for genetic variants, and phylogenetic analysis with previously published haplotypes was performed [16]. For this purpose, the sequences obtained in the present study and the haplotypes B1-B22 (KT693076-KT693094; EU544596; JQ663994; [16]) were trimmed to a uniform consensus length of 318 nucleotides. A Maximum Likelihood tree was constructed with MEGA X using the GTR+R+I substitution model [29]. *Hydatigera taeniaeformis* was used as an outgroup (KT693044). The haplotype diversity (Hd) was calculated using DnaSP 6.12.03 [30].

## 3. Results

### 3.1. Prevalence and Species Identification

In our sample set of 280 muskrats, metacestodes of five species of Taeniidae were found at different prevalence levels: *Echinococcus multilocularis* (41/280; prevalence = 14.6%), *Hydatigera kamiyai* (128/280; prevalence = 45.7%), *Taenia martis* (25/280; prevalence = 8.9%), *Taenia polyacantha* (14/280; prevalence = 5.0%), and *Versteria mustelae* (2/280; prevalence = 0.7%) (Table 1 and Table 2). Unambiguous morphological identification of the causative taeniid species was not possible for all specimens, especially of very young metacestodes. In those cases, and with a representative number of morphologically identified samples, molecular characterization of the parasites was conducted. In total, 98 *E. multilocularis* lesions were taken from 21 animals, 31 strobilocerci of *H. kamiyai*, 23 cysticerci of *T. martis*, 6 cysticerci of *T. polyacantha,* and 2 cysticerci of *V. mustelae* were identified by sequencing a fragment of the *cox1* gene. Obtained sequences were compared to reference sequences in GenBank. The acquired sequences of *E. multilocularis* varied in length from 300 to 1,590 base pairs, and no exchanges between them could be observed. The longest sequence with 1590 bp showed 100% identity with the European haplotypes E2, E4, and E5, which share the same *cox1* sequence [31]. All sequences of *H. kamiyai* (212–385 bp) had 98.96–100% identity with NC037071 [15]. *Taenia martis* sequences (405 bp) showed 99.75 –100% identity with AB731758 [15]. The *T. polyacantha* sequences (385 bp) were 100% identical to EU544581 [32], and *V. mustelae* (407 bp) showed 99.75% identity with AB732957 [15].

### 3.2. Infection Intensity

For *E. multilocularis*, 41/280 animals (14.6%) had metacestodes in the liver (Table 1). Seven out of those forty-one cases showed an advanced stage of disease with large parts of the liver replaced by metacestode tissue and involvement of neighboring organs. Thirty-four animals (82.9% of infected muskrats) had fertile metacestodes containing protoscoleces. The mean number of protoscoleces in the animals with fertile metacestodes was 311,714 (SE = 69,892); the highest number observed was 1,609,816 protoscoleces in one animal (Table 3). The mean weight of lesions per infected animal was 9.9 g (SE = 1.7), and the mean number of protoscoleces per gram of lesion was 19,899 (SE = 3240; see Table 3 for details of each age class).

For *H. kamiyai*, 128/280 (45.7%) muskrats had strobilocerci in the liver at a mean number of 3.2 (SE = 0.3) per infected animal. Twenty-five and 14 animals had cysticerci of *T. martis* (8.9%) and *T. polyacantha* (5.0%) in the abdominal cavity. In two animals, cysticerci of *V. mustelae* (0.7%) were found on the surface of the liver.

### 3.3. Genetic Diversity

In total, 98 discrete metacestode lesions of *E. multilocularis* from 21 infected animals resulted in an amplifiable *cox1* sequence of 300–1590 bp in length. Due to the different lengths, no exact statement can be made about possible genetic variants. No nucleotide exchanges could be detected between the sequences obtained, of which 33 were more than 1300 bp in length. The longest isolate was 100% identical to the European genotypes E2, E4, and E5 [31]. In the case of 23 *T. martis* isolates, the *cox1* fragments were 405 bp long and differed from each other in up to two nucleotides resulting in four haplotypes, the majority of the samples being 100% identical with AB731758 [15]. The six *T. polyacantha* and two *V. mustelae* sequences were all identical.

From a total of 31 strobilocerci of *H. kamiyai* (each from different hosts), 26 gave sequences of sufficient length of 396 bp for the analysis of the genetic diversity and comparison with already published haplotypes. In total, nine different haplotypes could be identified. Four of these haplotypes are already known from deposited sequences, and five have not been described and were submitted to GenBank (OP363933-OP363937; Table 4).

**Figure 2:**
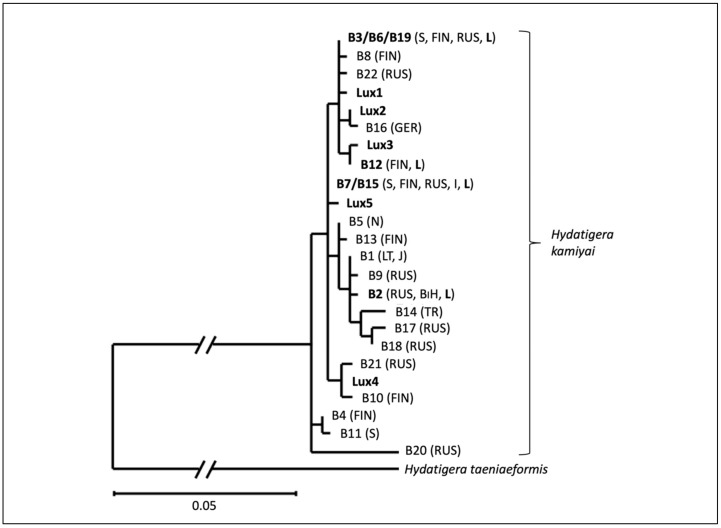
Maximum Likelihood analyses of 318 bp long *cox1* fragment of the known haplotypes of *Hydatigera kamiyai*. Haplotypes B1-B22 obtained from Lavikainen et al. (2016) and Lux1-Lux5 from the present study. Haplotypes found in Luxembourg are in bold. Countries of haplotype origin are in parentheses (abbreviation: BIH—Bosnia Herzegovina; FIN —Finland; G—Germany; J—Japan; I—Italy; L—Luxembourg; LT—Lithuania; N—Norway; R—Russia; S—Sweden). Outgroup sequence: *H. taeniaeformis* haplotype A1 (KT693044). Length of branches were shortened by 0.3.

For the phylogenetic analysis, the present sequences, as well as the reference sequences, had to be shortened to a common length of 318 bp. The shortening of the length of the 22 reference haplotypes B1–B22 [16] resulted in a reduction of the number of haplotypes to 19. This affected five of the original haplotypes. B3, B6, and B19 constituted a single haplotype with a shorter sequence, as did B7 and B15. The phylogram of the 318 bp long *cox1* fragment of the reference haplotypes and those found in the present study show that seven of the nine genetic variants found in Luxembourg cluster more closely together in the phylogram of *H. kamiyai* with all haplotypes known to date (Figure 2).

### 3.4. Risk Factors

**Figure 3:**
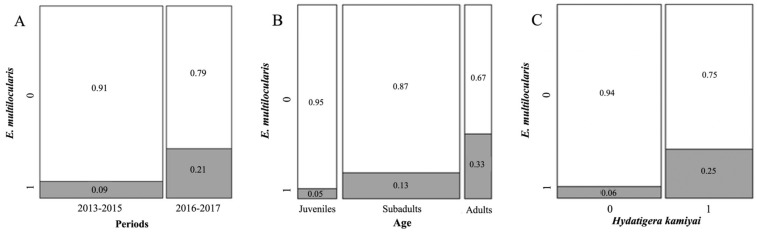
(**A**) Presence/absence of *E. multilocularis* in 2013–2015 (Period 1) and 2016–2017 (Period 2) of muskrats (n = 280) collected in North of Luxemburg. Evaluation was performed with Pearson’s Chi-squared test with Yates’ continuity correction. *X*^2^ = 13.380, df = 1, *p* < 0.001; (**B**) Presence/absence of *E. multilocularis* in class of age (juveniles, subadults, adults) of muskrats (n = 280) collected in North of Luxemburg, in period between 2013 and 2017. Evaluation was performed with Pearson’s Chi-squared test. *X*^2^ = 16.270, df = 2, *p* < 0.001; (**C**) Presence/absence of *E. multilocularis* and *H. kamiyai* in muskrats (n = 280) collected in North of Luxemburg in period between 2013 and 2017. Evaluation was performed with Pearson’s Chi-squared test with Yates’ continuity correction. *X*^2^ = 18.739, df = 1, *p* < 0.001.

Among the risk factors, we detected statistical differences between the frequency of *E. multilocularis* infections among periods of sampling with a higher frequency of infections in the second time period 2016/2017 (Figure 3A; *X*^2^ = 13.380, df = 1, *p* < 0.001), between the different muskrat age classes with an increasing frequency from juveniles to adults (Figure 3B; *X*^2^ = 16.270, df = 2, *p* < 0.001), and finally an increased frequency when in co-infection with *H. kamiyai* (Figure 3C; *X*^2^ = 18.739, df = 1, *p* < 0.001). The other factors did not present any significant difference in infection (*p* > 0.05).

**Figure 4:**
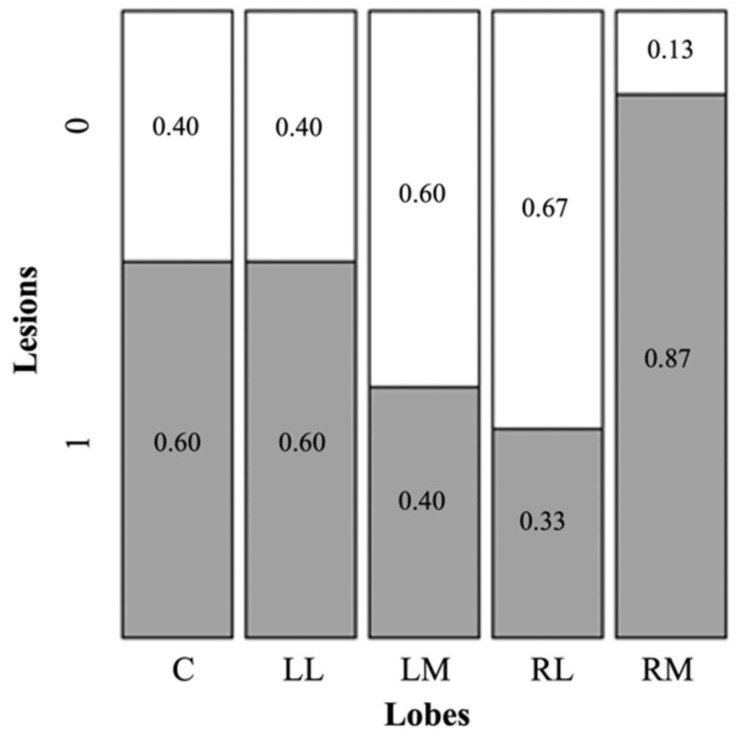
Presence/absence of lesions by *E. multilocularis* in liver lobes of muskrats (n = 15) collected in North of Luxembourg in period between 2013 and 2017. Evaluation was performed with Person’s Chi-squared test with simulated p-value (based on 2000 replicates). *X*^2^ = 10.606, df = NA, *p* = 0.036. Caudate ‘C’, Left Lateral ‘LL’, Left Medial ‘LM’, Right Lateral ‘RL’, Right Medial ‘RM’.

As for the homogeneity of lesions in the liver lobes, we detected a significantly uneven distribution in the various lobes (Figure 4; *X*^2^ = 10.606, df = 4, *p* = 0.036).

Confirming the preliminary findings, the model selection process identified a Generalized Linear Model with logit link including period, age class, and infection by *H. kamiyai* (Table 5; *p* < 0.001), with *E. multilocularis* infection in muskrats significantly correlated to the presence of *H. kamiyai* (Table 5, Figure 3C), to age class with a tendency of adult muskrats to be more infected than subadults and juveniles (Table 5, Figure 3B) and a difference between the two study periods with an increment in the likelihood of infection in the latter period (Table 5, Figure 3A). The origin of the animals did not contribute significantly to the model. Nonetheless, the model explained only a marginal observed variability (adjusted Nagelkerke *R*^2^ = 0.222), indicating that other factors might play a more relevant role in addition to the ones we analyzed.

### 3.5. Hepatic Distribution

The results showed that the right medial lobe was more likely to present lesions if compared with the other liver lobes (Table 6, Figure 4), particularly with the right lateral and left medial lobe (*p* < 0.005 and *p* < 0.01, respectively), whereas for the caudate and left lateral, we observed only a tendency to significance (*p* = 0.094). Specifically, lesions in the caudate and left lateral were more frequent than lesions in the right lateral and left medial, whereas, when compared to the right medial, caudate and left lateral appeared to have a lower frequency of lesions. No difference in the density of lesions (n lesions per gram) and density of protoscoleces (number of protoscoleces per gram) in the different lobes were detected (all comparisons by linear model using lobes as fixed factors; *p* > 0.05).

## 4. Discussion

The prevalence of *E. multilocularis* in muskrats in the present study (41/280; prevalence = 14.6%) was higher than the one reported in an earlier survey 2010–2012 from the same area, where 13 out of 147 animals (8.8%) were infected [34]. As there was also a significant increase in prevalence between the two periods of the present study (2013–2015 and 2016–2017), a trend toward increasing infection pressure was apparent. This is in accordance with the trend observed in definitive hosts (red foxes) in Luxembourg, based on data reported to the European Food Safety Authority (EFSA): in the period 2010–2013, 14.9% of 114 foxes were infected, in the period 2014–2017 it was 27.0% of 293 foxes (data processed from EFSA 2012; 2013; 2014; 2015; 2016; 2017; 2018). The prevalence estimates from muskrats in Luxembourg are comparable to those found in Belgium a decade earlier [14,35] but lower than those observed in southwestern Germany, where 28.9% of 702 muskrats were infected with *E. multilocularis* metacestodes in the period 1995–1997 (from 14.8% to 39.0% in different rivers and water bodies); more recent data from this area are not available [36]. Contrary to these highly endemic areas, muskrats were infected at a much lower prevalence (from 0.0% to 8.1%) in western France, the Netherlands, northern/eastern Germany, and Lithuania [10,11,12,13,37,38,39]. However, prevalence figures are difficult to compare when obtained in different periods, as the elimination of wildlife rabies and the rise of the fox population in the decade after 1990 resulted in far higher prevalence levels of *E. multilocularis* both in foxes and intermediate hosts in central Europe [40].

Despite being the most frequently infected intermediate host species, prevalence levels of metacestode infection in muskrats are generally lower than the prevalence of adult worm infection in foxes. However, where data from both muskrats and foxes are available from the same region and period, prevalence levels of both species are highly correlated, and muskrats have been suggested as a suitable indicator species for *E. multilocularis* endemicity where fox data are not available [35]. The data of the present study support this finding.

Muskrats show far higher prevalence levels than other competent intermediate host species in the same areas, such as common voles (*Microtus arvalis*) (1/914, prevalence = 0.11%, 0.003–0.61) and bank voles (*Myodes glareolus*) (1/23, prevalence = 4.3%, 0.1–21.9) [35], although this distinction becomes blurred when data from different regions and periods are pooled [8], and when prevalences of small arvicolines in micro-foci of intense transmission are considered [41,42]. As other arvicoline species are also highly susceptible to infection [2,43,44], this observation has been explained by the longer lifespan of muskrats, the larger and diversified food intake, and the semi-aquatic habitat, which is likely to facilitate the survival of *Echinococcus* eggs which are sensitive to desiccation and higher temperatures [45].

Based on susceptibility to infection, fertility of metacestodes, frequency, and predation rate by foxes, the common vole (*Microtus arvalis*) was convincingly proposed as the key intermediate host of *E. multilocularis* in central Europe [42]. Despite high prevalence levels, the role of muskrats in the lifecycle of *E. multilocularis* is less clear. An important feature of an efficient intermediate host is the regular predation by foxes [46]. Although foxes are certainly able to kill and feed on muskrats [47], the population size of muskrats is far smaller than that of small arvicolines, e.g., common voles, and their home ranges are restricted to the immediate vicinity of water bodies. Thereby, the contribution of muskrats to *E. multilocularis* transmission is likely to be restricted to specific habitats or special circumstances where, e.g., skinned muskrat carcasses are left in the environment or carcasses of trapped muskrats are used by hunters as bait for carnivores or wild boar [9,35] (Romig, personal observation). However, where foxes have access to muskrats, the large number of protoscoleces produced in the metacestodes has the potential to cause massive worm burdens in foxes. In this study, infected muskrats harbored a mean number of 312,000 protoscoleces, with a maximum of more than 1.6 million per animal. This is in contrast to common voles and bank voles in a highly endemic area, which harbored a mean number of 30,000 and 92,920 protoscoleces (with a maximum of 370,800 and 175,000, respectively) [42], and to wild-caught water voles with the highest reported number of protoscoleces of 451,540 [41]. The consumption of an infected muskrat by a suitable definitive host would result in a drastically higher worm burden and, therefore, higher egg excretion contaminating the environment. A further role of muskrats in the epidemiology of *E. multilocularis* could be the stabilization of the lifecycle, as the life span of muskrats is longer than that of small arvicolines [48]. In contrast to *Microtus* and *Arvicola* species, they do not show cyclic population dynamics and may therefore be able to carry metacestodes through periods of population breakdowns of other intermediate hosts [49].

In this study, muskrats were also infected with metacestodes of *Hydatigera kamiyai*, *Taenia martis*, *T. polyacantha,* and *Versteria mustelae*. Except for *T. polyacantha,* with foxes as definitive hosts, muskrats appear to be dead-end hosts for these cestodes, as they are not in the prey range of the respective definitive hosts (cats and mustelids). The frequency of *H. kamiyai* in this study (45.7%) is in a similar range as was reported from previous studies (under the species name *Taenia taeniaeformis*) in the Netherlands (44.8% [12]), southwestern Germany (48.1% [9]), and northern Germany (44.2% [10]; 42.3% [11]), while 23% of 130 animals were infected in northeastern Germany [13] and 80% of 30 animals in southern Poland [50]. This remarkably high and stable infection frequency across different periods and regions can be explained by the ubiquity and stable population of the most important definitive host in central Europe, the domestic cat. In contrast, the frequency of *T. martis* (8.9%), which likely reflects the different abundance of martens as definitive hosts, was higher than in the northern part of the Netherlands and northern/eastern Germany (0.0 to 6.1%) but lower than in the Southeast of the Netherlands (18.6%) and much lower than the 47.4% found in southwestern Germany [9,10,11,12,13]. *Taenia polyacantha* and *T. crassiceps*, both cestodes of foxes, are rare or absent in most published muskrat surveys. While *T. crassiceps* was also absent in our samples, *T. polyacantha* was unexpectedly frequent with a 5.0% prevalence, only comparable with an older study in southwestern (7.3%) and northeastern Germany (3.1%) [9,13]. Similarly, *V. mustelae* (0.7% in this study) had only been reported once from European muskrats in southwestern Germany at 4.8% prevalence [9]. In contrast, infection with *V. mustelae* (as *Taenia mustelae*) has been variously reported from muskrats in their native range in North America, where the American mink (*Neovison vison*), a muskrat predator, is a suitable definitive host [51]. However, the species allocation in North America is not certain, as various *Versteria* spp. seem to occur in that part of the world [52]. A fatal hyperinfestation with adult *V. mustelae* was recently reported from the endangered European mink (*Mustela lutreola*), so the occurrence of these larvae in muskrats may have a conservation aspect [53].

Although the significance level was not reached, our analysis suggests a tendency of an increased risk of becoming infected as the animal grows older. A positive correlation between age and infection was also previously observed both in muskrats (see, e.g., [35]) and *Arvicola terrestris* [41]. The longer life span of muskrats, compared to other Arvicolinae, is likely, in case of infection, to increase both the number of fertile metacestodes and the probability of reinfections.

Despite co-infections being a normal phenomenon in nature, the co-occurrence of *E. multilocularis* metacestodes with the larval stage of another cestode in the liver of the same individual has been described only once for *A. terrestris* in Central France [54] and in only one publication for *O. zibethicus*, the muskrat, in Western France [39]. In both cases, *E. multilocularis* was in association with what was then determined as *Taenia taeniaeformis*. Given the host species and geographical area, these records likely refer to *H. kamiyai* as well. In our study, we find a significant correlation between infection with both *E. multilocularis* and *H. kamiyai*. As the infection modes by accidental ingestion of eggs are identical, this association of the two parasites may be independent of our analyzed risk factors, such as age, and caused, for example, by food availability and habitat conditions. As a hypothesis, it also argues against an effective level of cross-protection by interspecific concomitant immunity.

In this study, we also addressed the question of whether there was a preferred liver lobe for the development of the *E. multilocularis* cysts. Although a previous study on *Echinococcus granulosus* highlighted no preference for a specific lobe of the mouse liver during the oncosphere migration process and the metacestode establishment and development [55], our data suggested that the *E. multilocularis* lesions in muskrats seemed to be preferentially localized in the right medial lobe of the liver. Nonetheless, we also showed that the lobe preference was not significant when the lobe mass was considered (i.e., no differences in density of lesions per mass of liver), and so the higher frequency of lesions was mostly caused by the larger mass of this lobe.

No genetic variation between *cox1* sequences was detected among 98 *E. multilocularis* samples from 21 different muskrats. Although, probably due to the long storage of the carcasses, obtained sequences varied in length, there were 33 sequences of >1300 bp, which did not show any nucleotide exchange between each other. The longest sequences were identical to the published *cox1* sequences of the haplotypes E2, E4, and E5, belonging to the European cluster of haplotypes [31]. This *cox1* variant is widespread and apparently frequent, having been found in France, Belgium, Germany, Slovakia [31], Poland [56,57], and Switzerland [58]. To further distinguish between the haplotypes E2, E4, E5, and others with a matching *cox1* sequence, the examination of further genes such as *nad2* and *cob* would be necessary. Genetic variability of *E. multilocularis* is known to be low within Europe, but the detection of only one haplotype in a considerable number of samples, although the samples originated all from a relatively small area, might indicate a recent founder event and subsequent population increase in the parasite in the study area.

The low variability of *E. multilocularis* is in stark contrast to the diversity among the 26 genetically analyzed samples of *H. kamiyai*. The analyzed sequences of only 318 bp gave nine different haplotypes, which is at the same level of diversity in this small study site of Luxembourg (Hd: 0.883 ± 0.031) as the published diversity of *H. kamiyai* based on 51 samples from large stretches of northern Europe, Russia, and the Balkan peninsula (Hd: 0.983 ± 0.021, when trimmed to the same sequence length) [16]. The present data, therefore, suggest that *H. kamiyai* in Europe (1) possesses a heterogenic genetic structure, and (2) the various haplotypes are distributed widely across the continent. This might indicate a long and undisturbed presence of this ubiquitous parasite in the distribution range. The genetic structure of wildlife cestode populations is poorly documented, and the present results contribute to our understanding of the population diversity of such parasites.

## 5. Conclusions

The results of this study show the presence of *E. multilocularis* and other taeniid species in muskrats in Luxembourg. The detected prevalence of *E. multilocularis* with 14.6% is far higher than in other competent intermediate hosts (0.11–4.3%) known from Europe; thus, muskrats could be used as sentinels for the presence of this particular parasite. Besides *E. multilocularis*, *Hydatigera kamiyai*, *Taenia martis*, *T. polyacantha,* and *Versteria mustelae* could be found. The most frequently detected parasite species was *Hydatigera kamiyai,* with 45.7% of the animals infected. Whereas the genetic diversity in *E. multilocularis* was very low (only one haplotype could be detected), the diversity of *H. kamiyai* with nine haplotypes was found to be far higher.

## Figures and Tables

**Table 1:** *Echinococcus multilocularis* infection for each class of weight and sex in muskrats collected in North Luxemburg from 2013 to 2017. **P%** prevalence expressed as percentage (N_i_, actual number of infected muskrats; N, number of necropsied carcasses) along with Wilson 95% CIs.

Weight Class in g (Estimated Age Class)	P%(N_i_/N) (95% CI)		
	♂	♀	Total
**201–600 (juvenile)**	**5.9** (2/34) (1.6–19.1)	**4** (1/25) (0.7–19.5)	**5.1** (3/59) (1.7–14)
**601–1000 (sub-adult)**	**13.9** (14/101) (8.4–22)	**12.8** (10/78) (7.1–22)	**13.4** (24/179) (9.2–19.2)
**1001–1400 (adult)**	**28** (7/25) (14.3–47.6)	**41.2** (7/17) (21.6–64)	**33.3** (14/42) (21–48.5)
**Total**	**14.4** (23/160) (9.8–20.7)	**15** (18/120) (9.7–22.5)	**14.6** (41/280) (11–19.3)

**Table 2:** Other cestode infections for each class of weight and sex in muskrats collected in North Luxemburg from 2013 to 2017. **P%** prevalence expressed as percentage (N_i_, actual number of infected muskrats; N, number of necropsied carcasses) along with Wilson 95% CIs.

Weight Class (g)	P% (N_i_/N) (95% CI)							
	*H. kamiyai*		*T. polyacantha*		*T. martis*		*V. mustelae*	
	♂	♀	♂	♀	♂	♀	♂	♀
**201–600**	**23.5** (8/34) (12.4–40)	**24** (6/25) (11.5–43.4)	**5.9** (2/34) (1.6–19.1)	**8** (2/25) (2.2–25)	**8.8** (3/34) (3.1–23)	**4** (1/25) (0.7–19.5)	**0** (0/34)	**0** (0/25)
**601–1000**	**40.6** (41/101) (32–50.4)	**53.8** (42/78) (43–64.5)	**3** (3/101) (1–8.4)	**2.6** (2/78) (0.7–9)	**7** (7/101) (3.4–14)	**9** (7/78) (4.4–17.4)	**0.9** (1/101) (0.2–5.4)	**1.3** (1/78) (0.2–7)
**1001–1400**	**72** (18/25) (52.4–86)	**76.5** (13/17) (53–90.4)	**12** (3/25) (4.2–30)	**11.8** (2/17) (3.3–34.3)	**24** (6/25) (11.5–43.3)	**5.9** (1/17) (1.1–27)	**0** (0/25)	**0** (0/17)
**Total**	**41.9** (67/160) (35–50)	**50.8** (61/120) (42–59.6)	**5** (8/160) (2.6–9.6)	**5** (6/120) (2.3–10.5)	**10** (16/160) (6.3–15.6)	**7.5** (9/120) (4–13.6)	**0.6** (1/160) (0.1–3.5)	**0.8** (1/120) (0.2–4.6)

**Table 3:** *Echinococcus multilocularis* reproduction for each class of weight in muskrats collected in North Luxemburg from 2013 to 2017. N_i_: number of infected animals.

Weight Class (g)	*Echinococcus multilocularis* Lesions			
	N_i_	N_i_ without Protoscoleces	N_i_ with Protoscoleces (Mean n Protoscoleces; SE; Range)	Mean Lesion Weight (g)
**201–600**	3	1	2 (4427; 2771; 1656–7198)	1.1 ± 0.9
**601–1000**	24	5	19 (160,353; 37,999; 1966–588,768)	6.5 ± 1.2
**1001–1400**	14	1	13 (580,210; 148,186; 5292–1,609,816)	17.5 ± 3.9
**Overall**	41	7	34 (311,714; 69,892; 1656–1,609,816)	9.9 ± 1.7

**Table 4:** Haplotypes (*cox1*) of *Hydatigera kamiyai* found in Luxembourg.

Haplotypes	n Isolates	Known Geographic Distribution	References
B2	3	Bosnia, European Russia	[16]
B3/B6/B19	3	Sweden, Finland, Russia, Poland	[16,33]
B7/B15	6	Sweden, Finland, Russia (Western Siberia), Italy	[16]
B12	1	Finland	[16]
Lux1	5	-	Present study
Lux2	4	-	Present study
Lux3	2	-	Present study
Lux4	1	-	Present study
Lux5	1	-	Present study

**Table 5:** Influence of *H. kamiyai* co-infection, muskrat age, and period (2013–2015 vs. 2016/2017) on presence/absence of *E. multilocularis* infections in muskrats (n = 280) collected in North of Luxemburg in period between 2013 and 2017. Results from a Generalized Linear Model with logit link are reported.

Variable	Estimated Coefficients	Std. Error	*Z*	*p* ^(d)^
Intercept *H. kamiyai* co-infection^(a)^ Age class^(b)^ *juveniles* Age class^(b)^ *adults* Period^(c)^ 2	−3.22 1.41 −0.50 0.82 1.12	0.44 0.42 0.66 0.42 0.37	−7.37 3.36 −0.76 1.96 3.01	<0.001 *** <0.001 *** 0.447 0.050 * 0.003 **

^(a)^ Absence of *H. kamiyai* was set as reference category. ^(b)^ Class of age of muskrat, considering their weight: juveniles < 600 g, subadults from 600 to 1000 g, adults >1000 g. Class of subadults was set as reference category. ^(c)^ Period considers samples collected from 2013 to 2015 (“period 1”) and from 2016 to 2017 (“period 2”). Period 1 was set as reference category. ^(d)^ *** *p* ≤ 0.001; ** *p* ≤ 0.01; * *p* ≤ 0.05.

**Table 6:** Lobe preference for the metacestodes settlement and their development in the liver of muskrats (n = 15) collected in North of Luxemburg in period between 2013 and 2017. Model was formulated using a binomial link to a generalized linear mixed-effects model.

Variable	Estimate	Std. Error	*Z*	*p* ^(b)^
Presence/Absence of Lesions by *E. multilocularis*				
Intercept Left Lateral^(a)^ Left Medial^(a)^ Right Lateral^(a)^ Caudate^(a)^	2.14 −1.66 −2.62 −2.96 −1.66	0.86 0.99 1.02 1.05 0.99	2.50 −1.68 −2.56 −2.82 −1.68	0.012 * 0.094 0.010 * 0.005 ** 0.094

^(a)^ Right median lobe was set as reference category. ^(b)^ ** *p* < 0.01; * *p* < 0.05.

## Data Availability

Data are contained in the present article.

## References

[1] Kern P., da Silva A.M., Akhan O., Müllhaupt B., Vizcaychipi K.A., Budke C., Vuitton D.A. (2017). The Echinococcoses: Diagnosis, clinical management and burden of disease. Adv. Parasitol..

[2] Romig T., Deplazes P., Jenkins D., Giraudoux P., Massolo A., Craig P.S., Wassermann M., Takahashi K., de la Rue M. (2017). Ecology and life cycle patterns of *Echinococcus* species. Adv. Parasitol..

[3] Niethammer J., Krapp F. (1982). Handbuch der Säugetiere Europas. Nagetiere II. Vol 2/I.

[4] Triplet P. CABI—Invasive Species Compendium-*Ondatra zibethicus* (Muskrat). https://www.cabi.org/isc/datasheet/71816.

[5] Brzeziński M., Romanowski J., Żmihorski M., Karpowicz K. (2010). Muskrat (*Ondatra zibethicus*) decline after the expansion of American mink (*Neovison vison*) in Poland. Eur. J. Wildl. Res..

[6] Skyrienė G., Paulauskas A. (2013). Distribution of invasive muskrats (*Ondatra zibethicus*) and impact on ecosystem. Ekologija.

[7] Romig T., Bilger B., Dinkel A., Merli M., Mackenstedt U. (1999). *Echinococcus multilocularis* in animal hosts: New data from Western Europe. Helminthologia.

[8] Oksanen A., Siles-Lucas M., Karamon J., Possenti A., Conraths F.J., Romig T., Wysocki P., Mannocci A., Mipatrini D., Torre G.L. (2016). The geographical distribution and prevalence of *Echinococcus multilocularis* in animals in the European Union and adjacent countries: A systematic review and meta-analysis. Parasit. Vectors.

[9] Loos-Frank B., Zeyhle E. (1981). Zur Parasitierung von 3603 Rotfüchsen in Württemberg. Z. Jagdwiss..

[10] Friedland T., Steiner B., Böckeler W. (1985). Prävalenz der Cysticercose bei Bisams (*Ondatra zibethica* L.) in Schleswig-Holstein. Z. Jagdwiss..

[11] Baumeister S., Pohlmeyer K., Kuschfeldt S., Stoye M. (1997). [Prevalence of *Echinococcus multilocularis* and other metacestodes and cestodes in the muskrat (*Ondatra zibethicus* L. 1795) in Lower Saxony]. Dtsch. Tierärztl. Wochs..

[12] Borgsteede F.H.M., Tibben J.H., van der Giessen J.W.B. (2003). The muskrat (*Ondatra zibethicus*) as intermediate host of cestodes in the Netherlands. Vet. Parasitol..

[13] Schuster R.K., Specht P., Rieger S. (2021). On the helminth fauna of the muskrat (*Ondatra zibethicus* (Linnaeus, 1766)) in the Barnim district of Brandenburg State/Germany. Animals.

[14] Mathy A., Hanosset R., Adant S., Losson B. (2009). The carriage of larval *Echinococcus multilocularis* and other cestodes by the muskrat (*Ondatra zibethicus*) along the Ourthe River and its tributaries (Belgium). J. Wildl. Dis..

[15] Nakao M., Lavikainen A., Iwaki T., Haukisalmi V., Konyaev S., Oku Y., Okamoto M., Ito A. (2013). Molecular Phylogeny of the Genus *Taenia* (Cestoda: Taeniidae): Proposals for the resurrection of *Hydatigera* Lamarck, 1816 and the creation of a new genus *Versteria*. Int. J. Parasitol..

[16] Lavikainen A., Iwaki T., Haukisalmi V., Konyaev S.V., Casiraghi M., Dokuchaev N.E., Galimberti A., Halajian A., Henttonen H., Ichikawa-Seki M. (2016). Reappraisal of *Hydatigera taeniaeformis* (Batsch, 1786) (Cestoda: Taeniidae) sensu lato with description of *Hydatigera kamiyai* n. sp.. Int. J. Parasitol..

[17] EFSA, ECDC (2018). The European Union summary report on trends and sources of zoonoses, zoonotic agents and food-borne outbreaks in 2017. Efsa J..

[18] Greenwood P.E., Nikulin M.S. (1996). A Guide to Chi-Squared Testing.

[19] Hope A.C.A. (1968). A Simplified monte carlo significance test procedure. J. Royal Stat. Soc. Ser. B Methodol..

[20] Patefield W.M. (1981). An Efficient method of generating random R × C tables with given row and column totals. J. Royal Stat. Soc. Ser. C Appl. Stat..

[21] Fox J. (2016). Applied Regression Analysis and Generalized Linear Models.

[22] Stroup W.W. (2013). Generalized Linear Mixed Models: Modern Concepts, Methods and Applications.

[23] R Core Team (2018). R Foundation for Statistical Computing.

[24] Bates D., Mächler M., Bolker B., Walker S. (2015). Fitting linear mixed-effects models using Lme4. J. Stat. Softw..

[25] Brown L.D., Cai T.T., DasGupta A. (2001). Interval estimation for a binomial proportion. Stat. Sci..

[26] Nakao M., Sako Y., Ito A. (2003). Isolation of polymorphic microsatellite loci from the tapeworm *Echinococcus multilocularis*. Infect. Genet. Evol..

[27] Hüttner M., Nakao M., Wassermann T., Siefert L., Boomker J.D.F., Dinkel A., Sako Y., Mackenstedt U., Romig T., Ito A. (2008). Genetic characterization and phylogenetic position of *Echinococcus felidis* Ortlepp, 1937 (Cestoda: Taeniidae) from the African lion. Int. J. Parasitol..

[28] Wassermann M., Aschenborn O., Aschenborn J., Mackenstedt U., Romig T. (2015). A sylvatic lifecycle of *Echinococcus equinus* in the Etosha National Park, Namibia. Int. J. Parasitol. Parasites Wildl..

[29] Kumar S., Stecher G., Li M., Knyaz C., Tamura K. (2018). MEGA X: Molecular evolutionary genetics analysis across computing platforms. Mol. Biol. Evol..

[30] Rozas J., Ferrer-Mata A., Sánchez-DelBarrio J.C., Guirao-Rico S., Librado P., Ramos-Onsins S.E., Sánchez-Gracia A. (2017). DnaSP 6: DNA sequence polymorphism analysis of large data sets. Mol. Biol. Evol..

[31] Nakao M., Xiao N., Okamoto M., Yanagida T., Sako Y., Ito A. (2009). Geographic pattern of genetic variation in the fox tapeworm *Echinococcus multilocularis*. Parasit. Int..

[32] Lavikainen A., Haukisalmi V., Lehtinen M.J., Henttonen H., Oksanen A., Meri S. (2008). A Phylogeny of members of the family Taeniidae based on the mitochondrial *cox1* and *nad1* gene data. Parasitology.

[33] Bajer A., Alsarraf M., Dwużnik D., Mierzejewska E.J., Kołodziej-Sobocińska M., Behnke-Borowczyk J., Banasiak Ł., Grzybek M., Tołkacz K., Kartawik N. (2020). Rodents as intermediate hosts of cestode parasites of mammalian carnivores and birds of prey in Poland, with the first data on the life-cycle of *Mesocestoides melesi*. Parasit. Vectors.

[34] Nicodemus S. (2012). *Echinococcus multilocularis* and Other Cestoda Larvae in Muskrat (*Ondarta zibethicus*) in Luxembourg. Bachelor’s Thesis.

[35] Hanosset R., Saegerman C., Adant S., Massart L., Losson B. (2008). *Echinococcus multilocularis* in Belgium: Prevalence in red foxes (*Vulpes vulpes*) and in different species of potential intermediate hosts. Vet. Parasitol..

[36] Romig T., Kratzer W., Kimmig P., Frosch M., Gaus W., Flegel W.A., Gottstein B., Lucius R., Beckh K., Kern P. (1999). An epidemiologic survey of human alveolar echinococcosis in Southwestern Germany. Römerstein Study Group. Am. J. Trop. Med..

[37] Mažeika V., Kontenytė R., Paulauskas A. (2009). New data on the helminths of the muskrat (*Ondatra zibethicus*) in Lithuania. Estonian J. Ecol..

[38] Seegers G., Baumeister S., Pohlmeyer K., Stoye M. (1995). *Echinococcus multilocularis*–metazestoden bei Bisamratten in Niedersachsen. Dtsch. Tierärztl. Wschr..

[39] Umhang G., Richomme C., Boucher J.-M., Guedon G., Boué F. (2013). Nutrias and muskrats as bioindicators for the presence of *Echinococcus multilocularis* in new endemic areas. Vet. Parasitol..

[40] Romig T., Craig P., Pawlowski Z. (2002). Spread of *Echinococcus Multilocularis* in Europe. Cestode Zoonoses: Echinococcosis and Cysticercosis.

[41] Burlet P., Deplazes P., Hegglin D. (2011). Age, season and spatio-temporal factors affecting the prevalence of *Echinococcus multilocularis* and *Taenia taeniaeformis* in *Arvicola terrestris*. Parasit. Vectors.

[42] Beerli O., Guerra D., Baltrunaite L., Deplazes P., Hegglin D. (2017). *Microtus arvalis* and *Arvicola scherman*: Key players in the *Echinococcus multilocularis* life cycle. Front. Vet. Sci..

[43] Woolsey I.D., Jensen P.M., Deplazes P., Kapel C.M.O. (2015). Establishment and development of *Echinococcus multilocularis* metacestodes in the common vole (*Microtus arvalis*) after oral inoculation with parasite eggs. Parasitol. Int..

[44] Woolsey I.D., Jensen P.M., Deplazes P., Kapel C.M.O. (2016). Peroral *Echinococcus multilocularis* egg inoculation in *Myodes glareolus*, *Mesocricetus auratus* and *Mus musculus* (CD-1 IGS and C57BL/6j). Int. J. Parasitol. Parasites Wildl..

[45] Veit P., Bilger B., Schad V., Schafer J., Frank W., Lucius R. (1995). Influence of environmental factors on the infectivity of *Echinococcus multilocularis* eggs. Parasitology.

[46] Hegglin D., Bontadina F., Contesse P., Gloor S., Deplazes P. (2007). Plasticity of predation behaviour as a putative driving force for parasite life-cycle dynamics: The case of urban foxes and *Echinococcus multilocularis* tapeworm. Funct. Ecol..

[47] Danell K. (1978). Population dynamics of the muskrat in a shallow Swedish lake. J. Animal. Ecol..

[48] Beer J.R., Truax W. (1950). Sex and age ratios in Wisconsin muskrats. J. Wildl. Manag..

[49] Boussinesq M., Bresson S., Liance M., Houin R. (1986). A new natural intermediate host of *Echinococcus multilocularis* in France: The muskrat (*Ondatra zibethicus* L.). Ann. Parasitol. Hum. Comp..

[50] Kowal J., Nosał P., Adamczyk I., Kornaś S., Wajdzik M., Tomek A. (2010). [The influence of *Taenia taeniaeformis* larval infection on morphometrical parameters of muskrat (*Ondatra zibethicus*)]. Wiad. Parazytol..

[51] Ganoe L.S., Brown J.D., Yabsley M.J., Lovallo M.J., Walter W.D. (2020). A review of pathogens, diseases, and contaminants of muskrats (*Ondatra zibethicus*) in North America. Front. Vet. Sci..

[52] Deplazes P., Eichenberger R.M., Grimm F. (2019). Wildlife-transmitted *Taenia* and *Versteria* cysticercosis and coenurosis in humans and other primates. Int. J. Parasitol. Parasites Wildl..

[53] Fournier-Chambrillon C., Torres J., Miquel J., André A., Michaux J., Lemberger K., Carrera G.G., Fournier P. (2018). Severe parasitism by *Versteria mustelae* (Gmelin, 1790) in the critically endangered European mink *Mustela lutreola* (Linnaeus, 1761) in Spain. Parasitol. Res..

[54] Pétavy A.-F., Tenora F., Deblock S. (2003). Co-occurrence of metacestodes of *Echinococcus multilocularis* and *Taenia taeniaeformis* (Cestoda) in *Arvicola terrestris* (Rodentia) in France. Folia Parasitol..

[55] Zhang R.-Q., Chen X.-H., Wen H. (2017). Improved experimental model of hepatic cystic hydatid disease resembling natural infection route with stable growing dynamics and immune reaction. World J. Gastroentero..

[56] Umhang G., Knapp J., Wassermann M., Bastid V., de Garam C.P., Boué F., Cencek T., Romig T., Karamon J. (2021). Asian admixture in European *Echinococcus multilocularis* populations: New data from Poland comparing EmsB microsatellite analyses and mitochondrial sequencing. Front. Vet. Sci..

[57] Karamon J., Stojecki K., Samorek-Pierog M., Bilska-Zając E., Rozycki M., Chmurzynska E., Sroka J., Zdybel J., Cencek T. (2017). Genetic diversity of *Echinococcus multilocularis* in red foxes in Poland: The first report of a haplotype of probable asian origin. Folia Parasitol..

[58] Laurimäe T., Kronenberg P.A., Rojas C.A.A., Ramp T.W., Eckert J., Deplazes P. (2020). Long-term (35 years) cryopreservation of *Echinococcus multilocularis* metacestodes. Parasitology.

